# MG53 attenuates nitrogen mustard‐induced acute lung injury

**DOI:** 10.1111/jcmm.16917

**Published:** 2022-02-24

**Authors:** Haichang Li, Lucia Rosas, Zhongguang Li, Zehua Bian, Xiuchun Li, Kyounghan Choi, Chuanxi Cai, Xinyu Zhou, Tao Tan, Valerie Bergdall, Bryan Whitson, Ian Davis, Jianjie Ma

**Affiliations:** ^1^ Department of Surgery The Ohio State University Columbus Ohio USA; ^2^ Department of Veterinary Biosciences The Ohio State University Columbus Ohio USA; ^3^ Department of Veterinary Preventive Medicine The Ohio State University Columbus Ohio USA

**Keywords:** acute lung injury, MG53, nitrogen mustard, oxidative stress

## Abstract

Nitrogen mustard (NM) is an alkylating vesicant that causes severe pulmonary injury. Currently, there are no effective means to counteract vesicant‐induced lung injury. MG53 is a vital component of cell membrane repair and lung protection. Here, we show that mice with ablation of MG53 are more susceptible to NM‐induced lung injury than the wild‐type mice. Treatment of wild‐type mice with exogenous recombinant human MG53 (rhMG53) protein ameliorates NM‐induced lung injury by restoring arterial blood oxygen level, by improving dynamic lung compliance and by reducing airway resistance. Exposure of lung epithelial and endothelial cells to NM leads to intracellular oxidative stress that compromises the intrinsic cell membrane repair function of MG53. Exogenous rhMG53 protein applied to the culture medium protects lung epithelial and endothelial cells from NM‐induced membrane injury and oxidative stress, and enhances survival of the cells. Additionally, we show that loss of MG53 leads to increased vulnerability of macrophages to vesicant‐induced cell death. Overall, these findings support the therapeutic potential of rhMG53 to counteract vesicant‐induced lung injury.

## 1 INTRODUCTION

Sulphur mustard (SM) and nitrogen mustard (NM) are alkylating agents known to cause severe damage to organs including the skin, eyes, lungs and nervous system, among which pulmonary toxicity is the major cause of death.^1^ Pulmonary toxicity involves complicated cellular events, including DNA damage, oxidative stress, acute injury and inflammation.^2^, ^3^, ^4^ In those who survive vesicant exposure, progressive inflammation often leads to pulmonary fibrosis and prolonged respiratory dysfunction.^5^, ^6^, ^7^, ^8^

Pulmonary exposure to SM/NM induces alkylating injuries with an acute and a chronic injury phase. The acute lung injury develops due to oxidative stress, lipid peroxidation and liberation of inflammatory mediators.^9^, ^10^, ^11^ The prolonged injury is mediated by chronic inflammation and fibrotic remodelling in the upper and small airways.^12^, ^13^ Despite many studies investigating the mechanism of mustard‐induced lung injury, current therapies to treat mustard poisoning are mostly palliative.^4^, ^5^, ^6^, ^7^, ^8^, ^14^, ^15^, ^16^, ^17^, ^18^, ^19^ Therapeutic means that mitigate acute oxidative stress, harness inflammation and restore cell membrane integrity could potentially alleviate the deleterious impact of vesicant and other environmental insults in the lung.

MG53 is a member of the TRIM protein (TRIM72), which functions as an essential component of plasma membrane repair.^20^, ^21^ MG53 knockout mice (*mg53* ^−/−^) develop pulmonary pathology due to defective membrane repair.^22^ We have shown that the recombinant human MG53 protein (rhMG53) when administered either intravenously (IV) or via aerosol has the ability to effectively mitigate lung ischaemia‐reperfusion injury, lipopolysaccharide‐induced inflammation and porcine pancreatic elastase (PPE)‐induced emphysema in rodents and pigs.^23^ In addition to membrane repair, recent studies demonstrate an anti‐inflammatory function of MG53 in dampening NF‐kB signalling, and knockdown of MG53 leads to hyper‐inflammation in human macrophages.^24^, ^25^

Herein, we provide data to support the physiologic function of MG53 in lung protection and the potential therapeutic value of rhMG53 to treat vesicant‐induced lung injury. When compared to wild‐type littermates, *mg53* ^−/−^ mice display an exacerbated lung injury with a more severe lung dysfunction following exposure to NM. Additionally, we show that intravenous administration of rhMG53 to wild‐type mice after exposure to NM can mitigate the adverse effects of NM‐vesicant lung injury.

## 2 MATERIALS AND METHODS

### 2.1 Regents and recombinant human MG53 protein (rhMG53)

NM (Mechlorethamine hydrochloride) was purchased from Sigma‐Aldrich Chemicals Co. (St. Louis, MO). rhMG53 protein was purified from *E*. *coli* fermentation as described previously.^26^

### 2.2 Animals and animal treatment

All animal care and usage were done in accordance with federal policies and guidelines and approved by the Ohio State University's IACUC. *mg53* ^−/−^ mice, tPA‐MG53 and their *wild*‐*type* littermates were bred and generated as previously described.^20^, ^27^ Mice were anaesthetized by intraperitoneal (IP) injection of ketamine and xylazine (80 mg/kg:10 mg/kg, respectively) and then were treated with PBS or NM (0.125 mg/kg) by intratracheal instillation following the protocol as described previously with minor modification.^2^ NM was prepared immediately before administration. All procedures were performed in a designated room with chemical hood strictly following OSU Environment and Health and Safety guidelines. rhMG53 (2 mg/kg) in saline or saline alone was administered by daily IV injection right after NM treatment for five days.

### 2.3 Cells, cell culture and stress treatment

Human bronchial epithelial cells (B2B) and THP‐1 cells were purchased from the American Type Culture Collection (ATCC). The B2B and THP‐1 cells were grown in RPMI 1640 medium supplemented with 10% FBS, 100 U/ml penicillin and 100 μg/ml streptomycin at 37°C in the presence of 5% CO_2_. The sh‐MG53‐knockdown THP‐1 cells were created and cultured as described previously.^24^ Primary porcine aortic endothelial cells (PAoEC) were isolated as previously described^28^ and cultured in MEM containing 10% FBS, 2 mM glutamine, 100 U/ml penicillin and 100 µg/ml streptomycin (Lonza) with bovine brain extract at 37°C in the presence of 5% CO_2_.

### 2.4 Apoptosis assay and ROS measurement

Cell apoptosis was investigated by dual staining with Alexa Fluor 488 annexin V and propidium iodide (PI) (Invitrogen Cat# V13241) following the manufacture protocol. Briefly, PAoEC and B2B cells were seeded in 6‐well plates, cultured for 24 h and then incubated with 10 µM (for PAoEC)/20 µM (for B2B cells) NM or BSA control for 4 h, washed and incubated with BSA or rhMG53 (10 µg/ml) for another 20 h. Cells were detached by 0.25% trypsin‐EDTA solution and washed with PBS for 1 time, and Annexin V and PI staining were performed for FACS analysis and analysed as described previously.^29^

Cellular‐reactive oxygen species (ROS) production was measured using a ROS Detection Assay Kit (Abcam Cat#ab113851) according to manufacture instructions. Briefly, cells were seeded in 6‐well plates, cultured for 24 h and then incubated with 10 µM (for PAoEC)/20 µM (for B2B cells) NM or BSA control for 4 h, and washed with PBS, incubated with BSA or rhMG53 (10 µg/ml) and cultured for another 20 h. Cells were washed, stained and analysed with DCF staining. The intensity of red fluorescence was detected by Guava EasyCyte™ System, and images were taken by confocal microscope.

### 2.5 Lung function measurement

Mice were anaesthetized by intraperitoneal (IP) injection of diazepam (17.5 mg/kg) followed by ketamine (80 mg/kg), and lung mechanics were assessed as previous described.^30^ Briefly, mouse was mechanically ventilated on a computer‐controlled flexiVent FX piston ventilator (SciReq; Montreal, Canada), with a tidal volume of 10 ml/kg at a frequency of 200 breaths/minute, against 2–3 cmH_2_O PEEP, as in our previous studies. Following two total lung capacity manoeuvres to standardize volume history, basal airway resistance and dynamic lung compliance were measured by the forced oscillation technique.^31^

### 2.6 Antibodies and western blotting

Primary antibodies used in this study are as follows: anti‐cleaved caspase‐3 (Cell Signaling Technologies) and anti‐GAPDH (Santa Cruz Biotechnology). Total protein extractions were prepared and subjected to immunoblot analysis as described previously.^32^, ^33^ Briefly, after blocking, membranes were incubated with relevant antibodies and probed with corresponding HRP‐conjugated secondary antibodies (Cell Signaling Technologies). All films were developed with ECL‐Plus regents (GE healthcare) and imaged using ChemiDoc^TM^ Gel Imaging System (Bio‐Rad).

### 2.7 Cell membrane injury assay and confocal microscopy

For membrane repair assay, B2B cells were transfected with GFP‐MG53 and then subjected to microelectrode penetration‐induced acute cell membrane injury, and the data were analysed as previously described.^20^

### 2.8 Histology and immunofluorescent staining

Histology and immunofluorescent staining were performed as previously described.^32^, ^33^ Briefly, tissues were dissected from experimental animals and then fixed in 4% paraformaldehyde (PFA) overnight at 4°C. After fixing, samples were washed three times for 5 min with 70% ethanol. Washed samples were processed, embedded in paraffin. 4 μm thick paraffin sections were cut. Cells were fixed with 4% PFA.

### 2.9 Statistical analysis

All data are expressed as means ± standard error of mean (SEM). Statistical evaluation was conducted using the student's *t* test and by ANOVA for repeated measures. A value of *p* < 0.05 was considered statistically significant.

## 3 RESULTS

### 3.1 Mg53^−/−^ mice are more susceptible to NM‐induced lung injury

**FIGURE 1:**
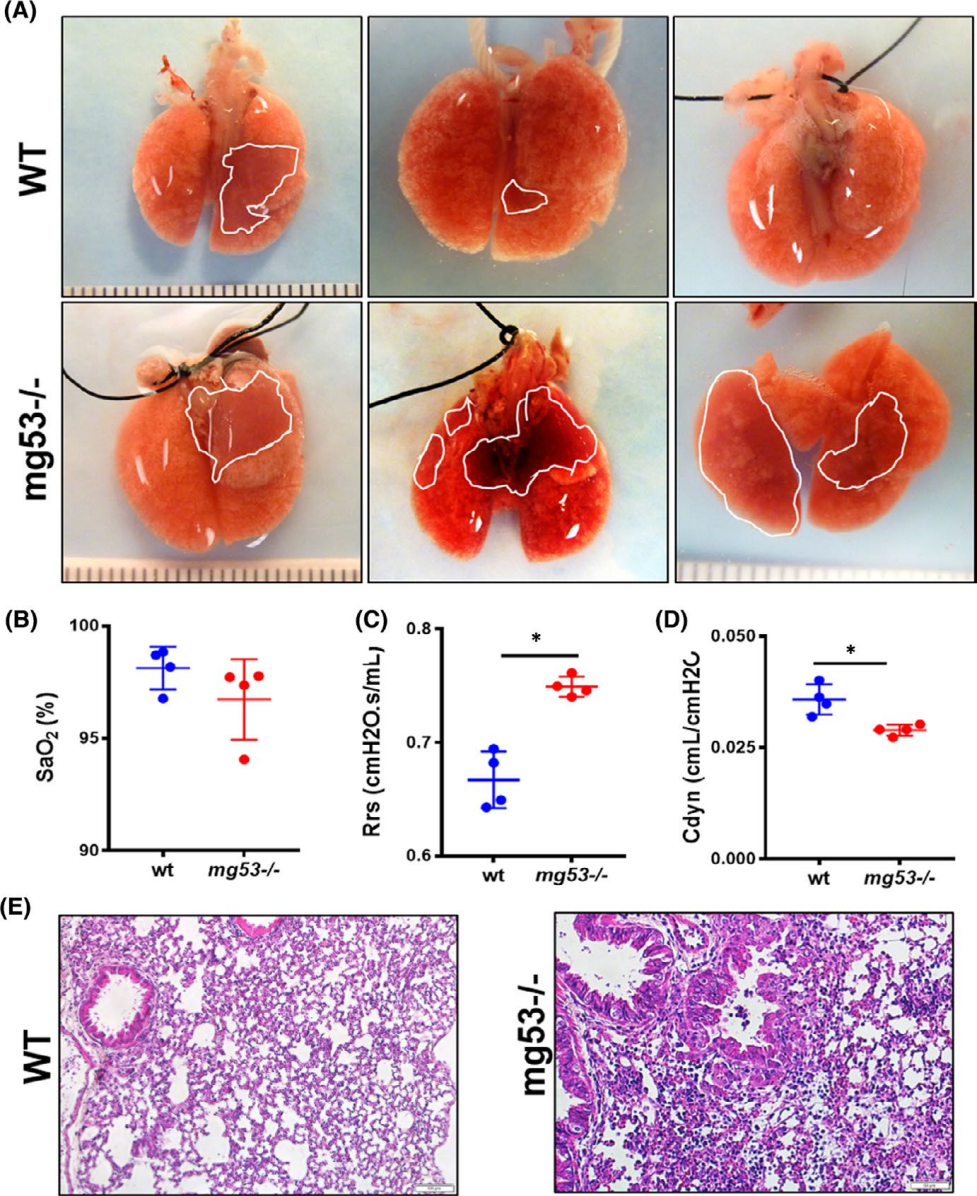
Knockout of MG53 enhances NM‐induced lung injury. Mice were treated by intratracheal instillation of either 50 µl NM (0.125 mg/kg in saline) or 50 µl saline alone. After 5 days of exposure, the lung function was examined, and mice were euthanized for lung tissue section preparation. (A) Representative images of *wild*‐*type* (upper panels) and *mg53* ^−/−^ (low panels) lung subjected NM exposure. Quantification of the lung function with blood oxygen saturation level (SaO_2_) (B), and resistance (Rrs) (C) and dynamic compliance (C_dyn_) (D). (E) Histological analyses revealed massive necrosis with increased infiltration of immune cells in the *mg53* ^−/−^ lung (right panel). These data demonstrate that ablation of MG53 renders the mice more susceptible to NM‐induced lung injury. *p < 0.05 for the indicated group [Colour figure can be viewed at wileyonlinelibrary.com]

To understand the physiological role of MG53 in protection against vesicant‐induced lung injury, we administered NM (0.125 mg/kg) intratracheally (IT) to *mg53* ^−/−^ mice and *wild*‐*type* littermate controls, according to the protocol developed by Laskin and colleagues.^2^ At 5 days post‐NM exposure, more severe lung damage was observed in *mg53* ^−/−^ mice than in *wild*‐*type* mice (Figure 1A). While carotid arterial oxygen saturations (SaO_2_) only showed a marginal difference between *wild*‐*type* and *mg53* ^−/−^ mice (Figure 1B), significant elevations of airway resistance (R_rs_, Figure 1C) and compromised lung dynamic compliance (C_dyn_, Figure 1D) were observed in the *mg53* ^−/−^ mice. Histological analysis revealed massive parenchymal necrosis with increased infiltration of immune cells in the *mg53* ^−/−^ lung (Figure 1E).

These observations are consistent with our recent findings that *mg53* ^−/−^ mice experience worsened morbidity and delayed recovery compared to wild‐type mice in a non‐lethal infection with influenza virus, correlating with increased inflammatory pathology in the lungs of the *mg53* ^−/−^ mice.^24^ Together, these data demonstrate that genetic ablation of MG53 renders the mice more susceptible to vesicant‐induced lung dysfunction and inflammation.

### 3.2 rhMG53 mitigates NM‐induced lung injury in mice

**FIGURE 2:**
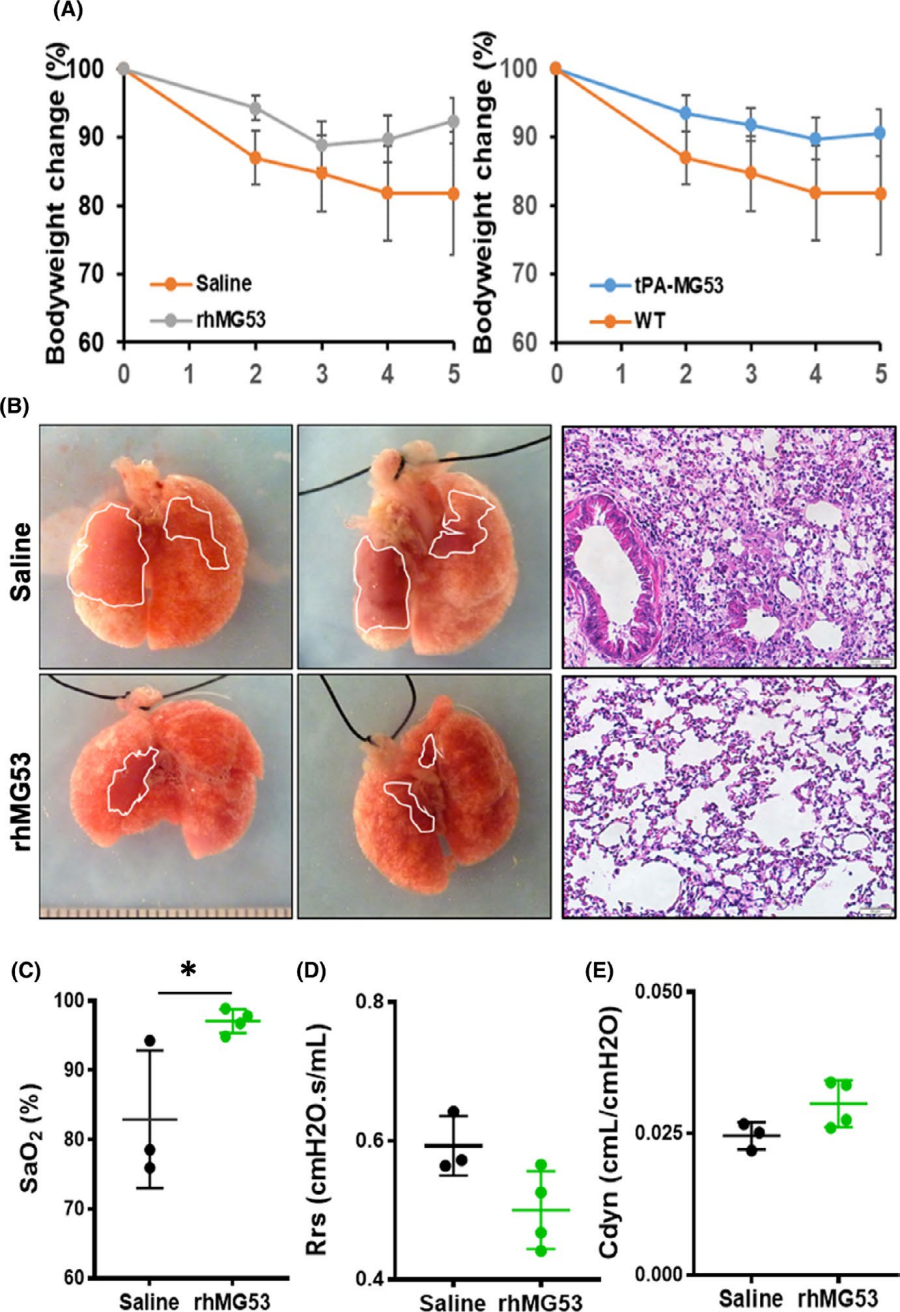
rhMG53 mitigates NM‐induced lung injury. Mice were treated by intratracheal instillation of either 50 µl NM (0.125 mg/kg in saline) or 50 µl saline alone. After exposure, the mice were weighted daily for up to 5 days post‐exposure (A). (B) Representative images (left panel) and HE staining (right panel) of saline control and rhMG53‐treated (IV, 2 mg/kg) lungs subjected to NM exposure. After 5 days of exposure, the lung function was examined, and mice were euthanized for lung tissue section preparation. Quantification of the lung function with blood oxygen saturation level (SaO_2_) (C), and resistance (Rrs) (D) and dynamic compliance (C_dyn_) (E). *p < 0.05 for the indicated group [Colour figure can be viewed at wileyonlinelibrary.com]

We recently reported that recombinant human MG53 protein (rhMG53) protects mice from lethal influenza virus infection and could function as a therapeutic to treat inflammation‐driven infectious diseases.^25^ To determine whether rhMG53 has similar protective effects against chemical lung injury, C57BL/6J mice (10 weeks age) were exposed to NM (0.125 mg/kg, IT) to induce lung injury. Mice were divided into two groups, one receiving tail vein administration of 2 mg/kg rhMG53 protein right after NM exposure (and then daily thereafter), and the others receiving saline as control at the same frequency with analysis conducted in a blinded manner. Mice receiving saline showed progressive decline of body weight, which was mitigated by rhMG53 treatment during the 5‐day observation period (Figure 2A, *left*). Out of the 4 mice in the control group, one died on day 2, while all rhMG53‐treated mice survived the 5 days observation period.

We have generated a transgenic mouse model with sustained elevation of MG53 level in the bloodstream (tPA‐MG53). Transgenic mice with baseline overexpression and secretion of increased levels of MG53 in the bloodstream (tPA‐MG53) live a healthier and longer life than littermate wild‐type mice. Western blot confirmed ~100‐fold elevation of MG53 in serum from the tPA‐MG53 mice compared with *wild*‐*type* littermates tPA‐MG53 mice display remarkable wound‐healing capacity and improved injury repair and regeneration.^34^ Compared with the *wild*‐*type* mice, the tPA‐MG53 mice experienced less weight loss after NM treatment (Figure 2A, *right*).

At the gross level, lung injury appeared less severe in rhMG53‐treated mice compared with the control group 5 days after NM exposure (Figure 2B). Even with the limited number of animals, we observed that rhMG53 treatment led to improved oxygen saturation (SaO_2_, Figure 2C) and trended towards increased dynamic lung compliance (C_dyn_, Figure 2D) and reduced airway resistance (R_rs_, Figure 2E).

### 3.3 NM exposure impairs MG53‐mediated membrane repair in lung epithelial cells

**FIGURE 3:**
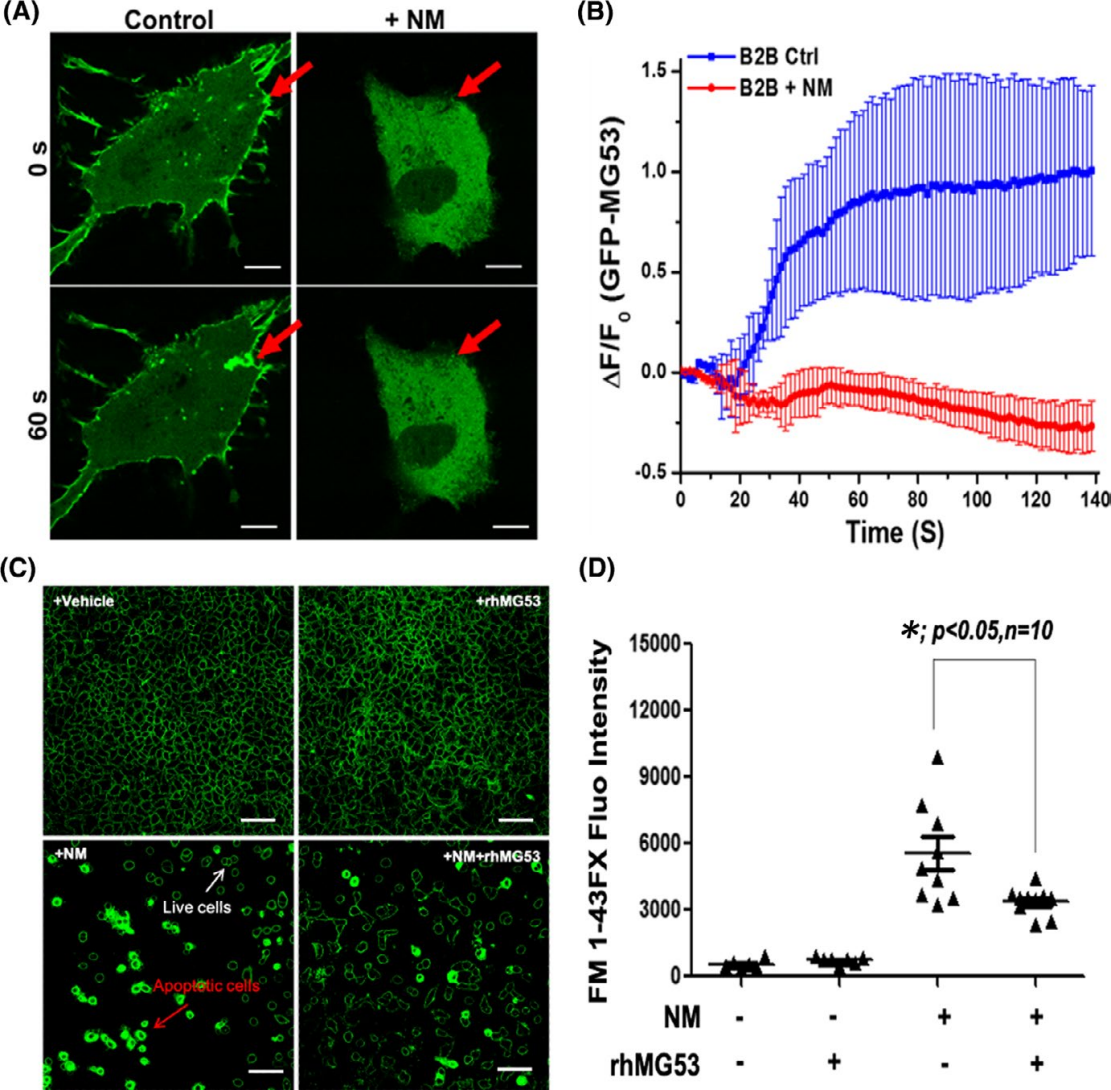
rhMG53 attenuates NM‐induced membrane damage in human lung epithelial (B2B) cells. (A) The B2B cells were transfected with GFP‐MG53. At 24 h after transfection, confocal fluorescence live‐cell imaging was conducted with control B2B cells and those treated with 20 µM NM, which then were injured by penetration of a microelectrode. The B2B cells show rapid translocation of GFP‐MG53‐containing intracellular vesicles towards the acute plasma membrane injury site following penetration of a microelectrode. Upper panel—cell image taken immediately after injury; lower panel—image taken 60 s after NM injury. Arrow shows the microelectrode injury site. Scale bar, 5 µm. (B) Time course of GFP‐MG53 accumulation at the injury sites following microelectrode penetration in NM‐treated B2B cells. (C) rhMG53 treatment suppresses FM1‐43FX dye entry in NM‐treated B2B cells following incubation with or without rhMG53. Scale bar, 25 µm. (D) Quantification of FM1‐43 dye entry in panel c (*n* = 10) [Colour figure can be viewed at wileyonlinelibrary.com]

To understand the mechanism that underlies MG53’s role in protection against NM‐induced lung injury, we conducted in vitro membrane repair assay as described previously.^20^, ^22^ Human lung epithelial (B2B) cells were transfected with GFP‐MG53 and treated with BSA vehicle or 10 µM NM. At 24 h after transfection, confocal fluorescence live‐cell imaging was conducted. As shown in Figure 3A, there were remarkable changes in the subcellular distribution of GFP‐MG53 in B2B cells treated with NM. GFP‐MG53 shows a plasma membrane localization pattern in control conditions, which was expected for the membrane repair‐patch function of MG53 (as observed in many other cell types^20^, ^26^, ^32^). However, GFP‐MG53 was mostly present in soluble and aggregated forms in the cytoplasm of B2B cells treated with NM.

Microelectrode poking induces acute injury to the plasma membrane of B2B cells, resulting in rapid translocation and accumulation of GFP‐MG53 at the site of injury for repair‐patch formation. The repair patch remained stable over the 2 min observation period in control cells not exposed to NM (Figure 3B, green). Remarkably, B2B cells treated with NM displayed dysfunctional GFP‐MG53 movement following microelectrode induced membrane injury (Figure 3B, red). While there was an initial small accumulation of GFP‐MG53 at the injury site within the first 30 s after injury, the repair patch did not remain stable as this decreased within the 2 min recording period.

Using FM1‐43 dye entry as a direct measure of cell membrane integrity,^20^, ^26^ we found that exposure of B2B cells to NM caused more entry of FM1‐43 dye, which could be reduced by treatment with rhMG53 (5 µg/ml) (Figure 3C,D). These findings provide direct evidence that vesicant exposure of lung epithelial cells leads to disruption of cell membrane repair machinery.

### 3.4 rhMG53 protects lung epithelial as well as endothelial cells against NM‐induced injury

**FIGURE 4:**
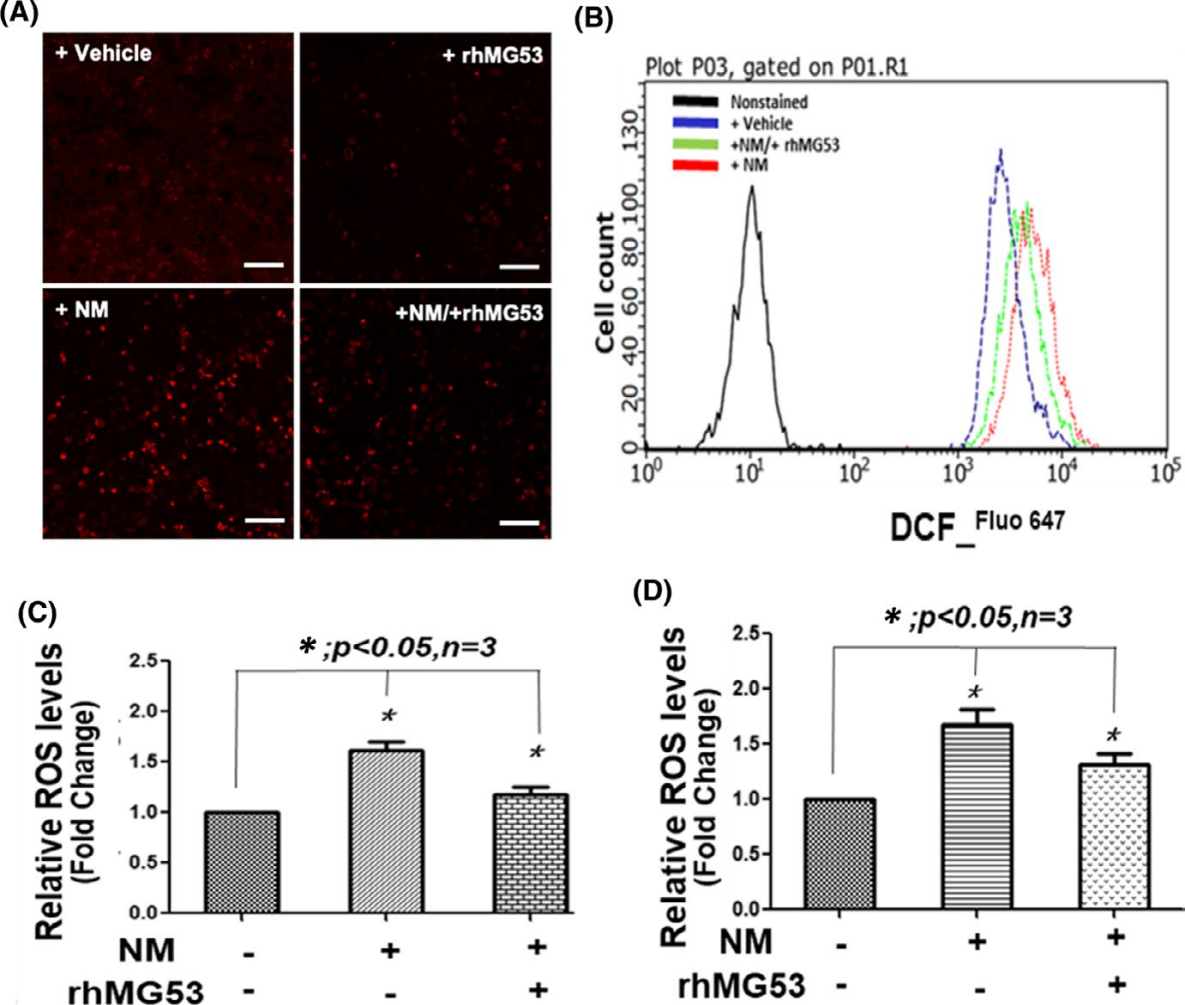
rhMG53 attenuates NM‐induced oxidative stress in human bronchial epithelial (B2B) porcine aortic endothelial (PAoEC) cells. The B2B cells and primary porcine aortic endothelial (PAoEC) cells were cultured for 24 h and treated with 10 µM (for PAoEC)/20 µM (for B2B cells) NM or BSA control for 4 h, and washed, incubated with BSA or rhMG53 (10 µg/ml) and cultured for another 20 h. Cells were stained and with DCF staining for ROS measurement. (A) Representative images for B2B with DCF staining. Scale bar, 25 µm. (B) Representative FACS analysis of B2B with DCF staining; (C) Quantification of B2B (FACS) for panel b (*n* = 3). (D) Quantification of PAoEC for analysis with DCF staining (FACS) (*n* = 3) [Colour figure can be viewed at wileyonlinelibrary.com]

Studies show that SM/NM exposure leads to elevation of cellular oxidative stress.^6^, ^10^, ^11^, ^35^, ^36^ We have demonstrated before that MG53’s membrane repair function is impaired when cells are subjected to chronic oxidative stress.^37^, ^38^ We next conducted a series of studies to quantify the potential protective role of rhMG53 in mitigating NM‐induced oxidative stress and injury to B2B cells. We used DCF fluorescent indicator to quantify intracellular ROS level and observed a significant increase in DCF fluorescence in B2B cells treated with 50 µM NM for 2 h (Figure 4A). FACS analysis was used to quantify the changes in ROS levels in B2B cells treated with NM (+saline) and NM (+rhMG53) (Figure 4B). rhMG53 treatment (5 µg/ml) significantly suppressed the NM‐induced elevation of ROS in B2B cells (Figure 4C).

**FIGURE 5:**
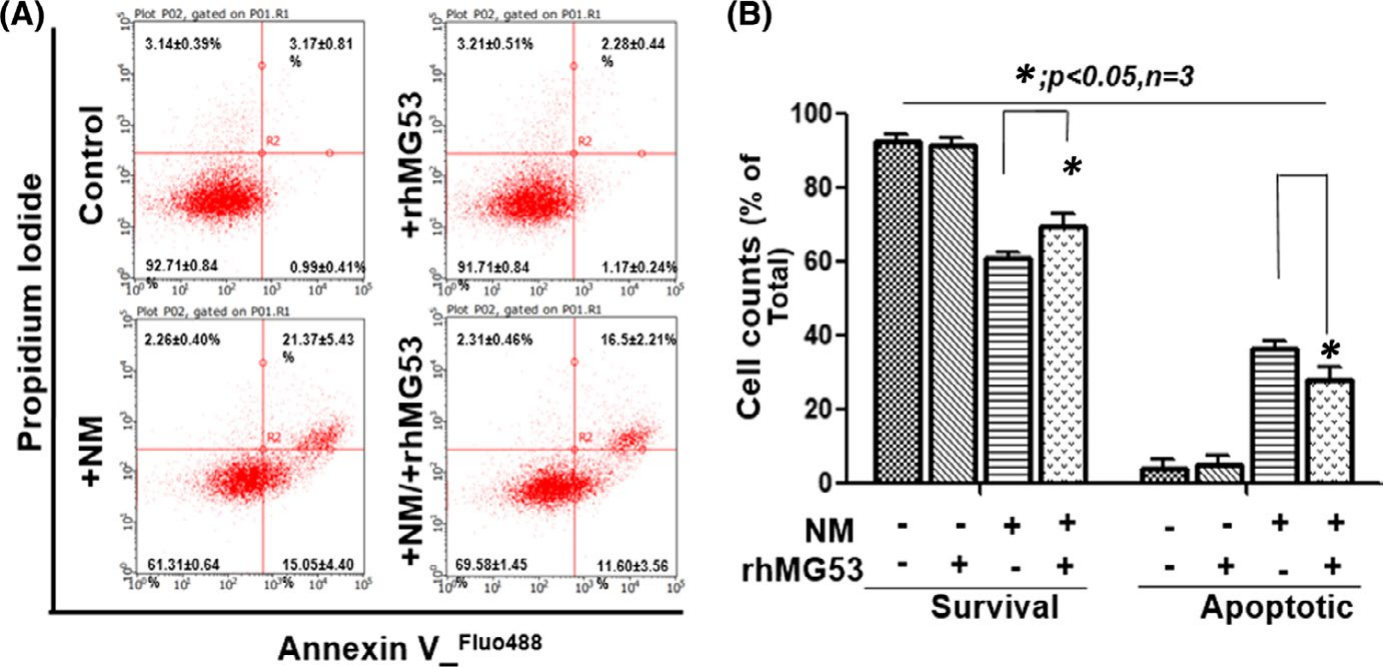
rhMG53 protects PAoEC cells from NM‐induced cell death. The PAoEC cells were cultured for 24 h, treated with 10 µM NM or BSA control for 4 h and then incubated with rhMG53 (10 µg/ml) or BSA for another 20 h. Cells were stained analysed with annexin V and PI staining and analysed. (A) Representative apoptosis analysis of PAoEC (FACS). (B) Quantification of PAoEC apoptosis for panel a (*n* = 3) [Colour figure can be viewed at wileyonlinelibrary.com]

Our laboratory has developed a protocol to isolate primary porcine aortic endothelial cells (PAoEC), which are widely used as an in vitro system to evaluate endothelial cell function (of either aortic or pulmonary tissues).^28^ Similar findings were also observed with PAoEC cells, where rhMG53 treatment ameliorated NM‐induced ROS generation (Figure 4D). To further quantify the degree of NM‐induced cell death, we conducted FACS analysis of PAoEC stained with propidium iodide (PI) and annexin V (Figure 5A). PAoEC cells that were annexin V‐positive and PI‐negative were defined as undergoing apoptotic cell death. As shown in Figure 5B, rhMG53 treatment improved survival of the PAoEC cells and reduced apoptotic cell death.

These findings provide a novel mechanism for NM‐induced tissue injury that involves oxidative stress‐mediated disruption of the cell membrane repair machinery. It also lays the foundation for the use of exogenous rhMG53 to boost the defence mechanism of these cells against vesicant‐induced tissue injury.

### 3.5 rhMG53 protects THP‐1 cells against NM‐induced injury

In addition to oxidative stress, vesicant exposure is known to cause sustained inflammation that contributes to the exacerbated and delayed lung injury. Thus, controlling inflammation is also important to combat vesicant‐induced lung injury.^2^, ^3^, ^4^, ^5^, ^7^, ^8^, ^14^, ^16^, ^17^, ^18^, ^39^ We recently reported that MG53 has an anti‐inflammatory role associated with tissue injury.^24^ We found that THP‐1 human macrophages express MG53 and that loss of MG53 leads to hyper‐inflammation due to activation of NF‐ĸB.

**FIGURE 6:**
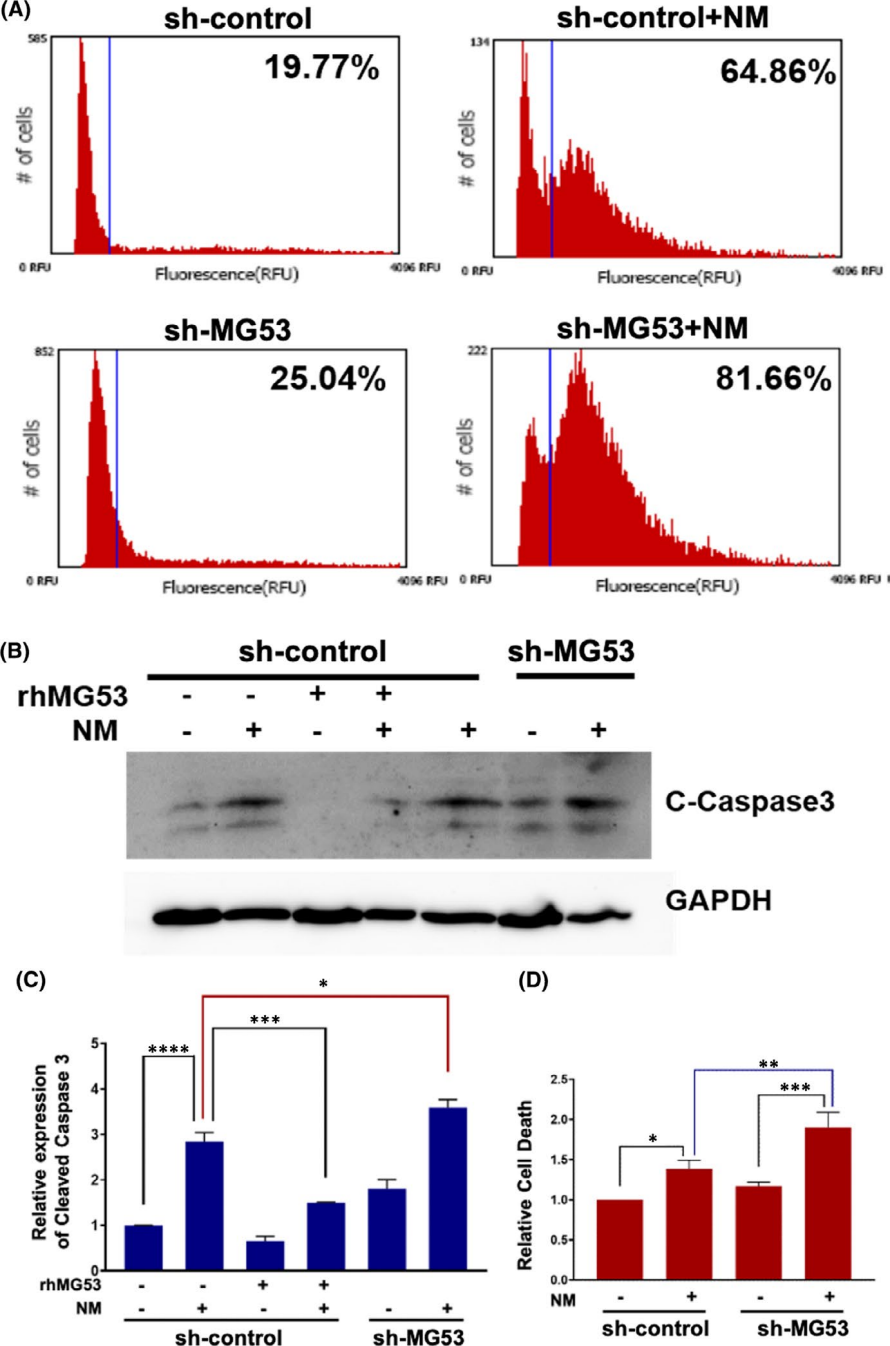
Knockout of MG53 results in an enhanced NM‐induced cell death in THP‐1 cells following NM exposure. The differentiated THP‐1 cells were cultured for 24 h, treated with 10 µM NM or BSA for 20 h and then incubated with vehicle or rhMG53 (10 µg/ml) for another 20 h. (A) Representative image of apoptosis assay for the sh‐control and sh‐MG53 THP‐1 cells. (B) THP‐1 protein lysates were analysed by Western blot using cleaved caspase‐3 and GAPDH antibody. (C) Relative expression of cleaved caspase‐3. (D) Qualification of cell death with PI staining. ****p < 0.0001, ^***^p < 0.001, ^**^p < 0.005, ^*^p < 0.05 for the indicated group. Data are presented as fold changes to control basal and as mean ± SEM (*n* = 6) [Colour figure can be viewed at wileyonlinelibrary.com]

We transfected THP‐1 cells with shRNA against MG53 to generate a MG53‐knockdown cell line (sh‐MG53) (Figure 6). As shown in Figure 6A, sh‐MG53 cells were more susceptible to NM‐induced cell death, compared with sh‐scramble THP‐1 cells. Western blot showed that NM‐induced activation of caspase‐3 in THP‐1 cells could be reduced by the addition of exogenous rhMG53 (Figure 6B,6C). PI staining showed enhanced cell death with MG53‐knockdown in THP‐1 cells following NM exposure (Figure 6D). Thus, MG53 can protect against NM‐induced macrophage cell death, thereby enhancing the resolution of early phase of lung injury. These findings support the anti‐inflammatory function of MG53 associated with vesicant exposure.

## 4 DISCUSSION

MG53 plays an essential role in cell membrane repair and pulmonary protection.^20^, ^21^ MG53 also has anti‐inflammatory function associated with chronic injury, sepsis and viral infection.^24^, ^25^, ^40^ In this study, we demonstrated that knockout of MG53 causes more severe lung injury and lung dysfunction following exposure to NM. Previously, we have demonstrated pulmonary pathology with the *mg53* ^‐/‐^ mice, which may be the underlying contributor to the increased susceptibility of the lung to NM‐induced injury.^22^ Conversely, transgenic mice with increased levels of MG53 in the bloodstream were resistant to NM‐induced tissue injuries, indicating that circulating MG53 provides critical pulmonary protection. Systemic exogenous administration of rhMG53 mitigated the adverse effects of NM‐vesicant lung injury. Live‐cell imaging revealed that exposure of lung epithelial cells with NM disrupts the intrinsic cell membrane repair ability of MG53, whereas the exogenous rhMG53 dampens oxidative stress caused by NM in both lung epithelial and endothelial cells. The loss of MG53 in cultured THP‐1 cells lead to their increased vulnerability to vesicant‐induced cell death. Together, these findings support the dual role of MG53 as a tissue‐repair molecule with anti‐inflammation function to protect the lungs against vesicant‐induced injuries. Targeting alveolar membrane injury repair may offer an effective means to rescue the damaged lung in the acute phase of injury and to prevent the progression into the prolonged injury phase.

Through live‐cell imaging, we demonstrated that, in lung epithelial cells exposed with NM, GFP‐MG53 becomes immobilized and is incapable of translocation to the membrane injury site for repair‐patch formation. These findings provide direct evidence that vesicant exposure of lung epithelial cells leads to disruption of cell membrane repair machinery.

Accumulating evidence suggests that the cytotoxic mechanism associated with mustard exposure contributes to oxidative stress, which induces damage to the lung.^6^, ^10^, ^11^, ^35^, ^36^ Previously, we have demonstrated that MG53’s membrane repair function is impaired when cells are subjected to chronic oxidative stress.^37^, ^38^ The preservation of cell integrity and mitigation of oxidative stress can provide added benefits to pulmonary protection. Using cultured lung epithelial (B2B) and endothelial (PAoEC) cells, we found that rhMG53 treatment can improve survival of the cells and reduce ROS levels upon exposure to NM. These findings provide evidence that NM‐induced tissue injury and oxidative stress can all be mitigated by MG53, laying the foundation for the use of exogenous rhMG53 to boost the defence mechanism of the lungs against vesicant‐induced injury.

In addition to oxidative stress, mustard gas exposure is known to induce infiltration of inflammatory cells and cytokines release in the lung that contributes to the exacerbated and delayed lung injuries.^2^, ^8^, ^9^, ^41^ Macrophages are known to play a role in both acute and chronic pulmonary pathologies. An imbalance of macrophage‐released pro‐inflammatory and anti‐inflammatory cytokines will aggravate acute lung injury and promote the development of lung toxicity.^2^, ^3^, ^5^, ^7^, ^8^, ^15^, ^41^ By flow cytometric analysis, we showed that THP‐1 cells with knockdown of MG53 are more susceptible to NM‐induced cell death, while rhMG53 treatment enhances cell survival after NM exposure. Preservation of macrophage integrity by MG53 can add to the defence mechanism of the lungs during the early phase of NM exposure. In addition to maintenance of macrophage integrity, we recently showed that repetitive administration of rhMG53 could suppress the release of pro‐inflammatory cytokines, for example IL‐1β and IL‐6, in lung following influenza virus exposure in mice.^24^, ^25^ The anti‐inflammatory function of MG53 is associated with the control of intracellular Ca^++^ oscillation and NF‐ĸB activation. Future studies are required to dissect the signalling cascades that are associated with vesicant‐induced inflammation and the mechanisms that underlie MG53’s anti‐inflammatory role for the long‐term benefits of lung function following vesicant exposure.

In rodents and humans, MG53 is secreted by muscle cells and is present at low levels in blood in normal physiologic conditions.^26^, ^42^ Thus, a therapeutic approach that modulates endogenous MG53 levels/function or involves systemic administration of rhMG53 protein is potentially a safe biologic means to treat and prevent tissue damage, including vesicant‐induced multi‐organ injury. We have performed toxicological studies in rodents and dogs,^33^ which demonstrate that rhMG53 has broad safety, underscoring its promise as a potential therapeutic to treat multi‐organ injuries. Future studies testing the safety and efficacy of rhMG53 in large animal models of vesicant‐induced pulmonary injury represent an essential component for our effort in translating the basic findings with MG53 into human applications.

## CONFLICT OF INTEREST

J.M. and T.T. have equity interest in TRIM‐edicine, which develops MG53 for the treatment of human disease. Patents on the use of MG53 are held by Rutgers University and The Ohio State University.

## AUTHOR CONTRIBUTION

**Haichang Li:** Conceptualization (equal); Data curation (lead); Formal analysis (lead); Investigation (lead); Methodology (equal); Project administration (equal); Supervision (equal); Validation (lead); Writing‐original draft (lead); Writing‐review & editing (lead). **Lucia Rosas:** Data curation (lead); Formal analysis (lead); Investigation (equal); Methodology (equal); Supervision (equal); Validation (equal). **Zhongguang Li:** Data curation (equal); Formal analysis (equal); Validation (equal). **Zehua Bian:** Data curation (supporting); Investigation (equal); Visualization (supporting). **Xiuchun Li:** Data curation (equal). **Kyounghan Choi:** Data curation (equal). **Chuanxi Cai:** Data curation (equal). **Xinyu Zhou:** Data curation (equal); Formal analysis (equal). **Tao Tan:** Data curation (supporting). **Valerie Bergdall:** Project administration (equal); Writing‐review & editing (equal). **Bryan Whitson:** Conceptualization (equal); Project administration (equal). **Ian C. Davis:** Conceptualization (equal); Data curation (lead); Methodology (equal); Project administration (equal); Supervision (equal); Writing‐review & editing (equal). **Jianjie Ma:** Conceptualization (lead); Data curation (equal); Funding acquisition (lead); Investigation (lead); Project administration (lead); Supervision (lead); Writing‐original draft (equal); Writing‐review & editing (equal).

## Data Availability

The datasets generated during the current study are available from the corresponding author upon reasonable request.
